# Impact of the COVID-19 and War Migration on HIV/AIDS Epidemiology in Poland

**DOI:** 10.3390/jcm13144106

**Published:** 2024-07-13

**Authors:** Agnieszka Genowska, Dorota Zarębska-Michaluk, Miłosz Parczewski, Birute Strukcinskiene, Piotr Rzymski, Robert Flisiak

**Affiliations:** 1 Department of Public Health, Medical University of Bialystok, 15-295 Bialystok, Poland; 2 Department of Infectious Diseases and Allergology, Jan Kochanowski University, 25-317 Kielce, Poland; dorota1010@tlen.pl; 3 Department of Infectious, Tropical Diseases and Immune Deficiency, Pomeranian Medical University in Szczecin, 71-455 Szczecin, Poland; milosz.parczewski@pum.edu.pl; 4 Faculty of Health Sciences, Klaipeda University, LT-92294 Klaipeda, Lithuania; birute.strukcinskiene@ku.lt; 5 Department of Environmental Medicine, Poznań University of Medical Sciences, 60-806 Poznań, Poland; rzymskipiotr@ump.edu.pl; 6 Department of Infectious Diseases and Hepatology, Medical University of Bialystok, 15-540 Bialystok, Poland; robert.flisiak1@gmail.com

**Keywords:** HIV/AIDS, infection, mortality, time trends, COVID-19, war refugees

## Abstract

**Objectives**: This study analyzed trends in HIV/AIDS in Poland over the time period of 2009–2021 and the potential impact of COVID-19 and the migration of war refugees from Ukraine. **Methods**: Long-term trends were assessed by joinpoint regression using data from Polish HIV/AIDS registries. The HIV/AIDS burden was also compared before and during the pandemic and refugee migration. **Results**: In 2009–2021, the upward tendency in the rate of new HIV infections until 2017 and decrease after 2017 was accompanied by a downward trend in new HIV/AIDS diagnoses and mortality. From the pandemic’s beginning until March 2022, rates of new HIV/AIDS diagnoses dramatically decreased to later increase to pre-pandemic levels, which partially coincided with the wave of migration of refugees from Ukraine. **Conclusions**: Long-term analysis of HIV/AIDS in Poland showed a downward trend in new HIV/AIDS diagnoses and related mortality in 2009–2021. While the pandemic has reduced the number of detected HIV/AIDS cases, a subsequent increase in new HIV diagnoses in 2022 may be related to lifting the COVID-19 restrictions and war refugees’ migration. These observations have implications for the WHO European Region, seeking to end AIDS as a public health problem by 2030.

## 1. Introduction

Human immunodeficiency virus (HIV), a causative agent of acquired immunodeficiency syndrome (AIDS), continues to be associated with a substantial health burden, especially in middle- and low-income countries, and has already caused over 40 million deaths [1]. In 2022, nearly 40.0 million people had HIV globally, and 1.3 million new infection cases and 630 thousand AIDS-associated deaths were recorded. Although two-thirds of people with HIV/AIDS (over 25 million) are living in the WHO African Region [2], the ongoing viral transmission is observed in all countries globally, including developed areas and Eastern Europe [3,4]

Even though undiagnosed and untreated infection typically leads to the development of AIDS within 2–10 years [5,6], it may be effectively prevented by modern antiretroviral therapies (ART). Evolving antiretroviral treatment allows for complete suppression of viral replication, allowing for the reconstitution of immune function, lowering AIDS and non-AIDS-associated morbidity and mortality, decreasing chronic inflammation, and significantly diminishing the risk of HIV transmission [7].

In the last two decades, notable progress towards control of the HIV epidemic was observed with the introduction of combination preventive approaches, including pre-exposure prophylaxis, test-and-treat approach, and advances towards the universal and global roll-out of ART. Despite introducing novel diagnostic approaches, the progress in reducing late diagnoses (with lymphocyte CD_4_ count < 350 cells/µL or AIDS-defining condition and excluding recent HIV infection) has been suboptimal [8]. Joint United Nations Program on HIV/AIDS evolved over the years, providing goals to combat HIV/AIDS epidemics, introducing “3 by 5”, “15 by 15”, “90-90-90”, and “95-95-95” strategies [7,9]. The latter was introduced in 2020 and aims to diagnose at least 95% of all infected people, provide ART to at least 95% of those diagnosed, and achieve viral suppression for at least 95% of those treated by 2030. Despite these goals, the challenges remain, and many countries are still a long way from the current goal, especially from the testing perspective [9,10]. While adults receiving ART reach the 95% viral suppression target, long-term suppression has proven challenging, particularly among children and adolescents [11]. The multiregional, retrospective cohort study conducted in 31 countries demonstrated that 79% of adults and 64% of children and adolescents on ART were virally suppressed after one year of therapy, 72% and 62% after two years, and 65% and 59% after three years, indicating that the efforts to reach and sustain viral suppression must go further [11]. Moreover, the observed emergence and increased frequency of drug- and multidrug-resistant HIV strains may be of concern. However, this risk is being partially mitigated by the introduction of population therapies, with core agents being high genetic barrier integrase inhibitors such as dolutegravir or bictegravir [12].

According to a recent joint report by the European Centre for Disease Prevention and Control (ECDC) and the WHO Regional Office for Europe, more than 2.3 million people are affected by HIV in Europe, particularly in the eastern part, while late HIV diagnosis remains a challenge for most countries [13]. The COVID-19 pandemic has further negatively affected the situation because of the major disorder in the healthcare system and decreased social mobility, especially during the first waves of SARS-CoV-2 infections [14,15]. Moreover, case studies show that 4% of HIV-infected patients experienced an interruption of ART, and 6% could not collect ART due to mobility restrictions, while some European countries experienced shortages in HIV medicines [16,17,18]. This could be further complicated in Europe, particularly in Poland, due to the Russian war in Ukraine in February 2022 and the subsequent inflow of war refugees [19,20]. Since this aspect has not been studied so far, it is crucial to study the epidemiology and dynamics of HIV/AIDS transmission under the pressure of the pandemic and the early influence of war. A reliable assessment of the impact of war migration on the epidemiology of HIV/AIDS is possible due to the system of organizing medical care for refugees in Poland. The right to free health care services in Poland is available to people covered by health insurance (compulsory or voluntary) and family members of the insured person. In Poland, health insurance is the responsibility of the National Health Fund, to which the health insurance contributions must be transferred. The Law of 12 March 2022, effective from 24 February 2022, on assistance to war refugees from Ukraine grants Ukrainian citizens who came to Poland due to Russian aggression the right to health benefits, reimbursement of medicines, and provision of medical devices on the same terms as Polish citizens [21]. The only condition for providing medical care to war refugees is their registration in Poland. Free medical care also includes access to diagnosis and treatment of HIV. Patients with HIV/AIDS are treated under public health care.

This article aimed to analyze HIV/AIDS infections and mortality in Poland, trends in 2009–2021, and to assess the potential impact of the COVID-19 pandemic and the migration of war refugees from Ukraine on the epidemiology of HIV/AIDS in Poland.

## 2. Materials and Methods

### 2.1. Data Extraction

In the present population-based retrospective study, we used all new cases of HIV, new diagnoses of HIV/AIDS, and related mortality registered in Poland over 13 years (2009–2021). Moreover, to understand the possible influence of the COVID-19 outbreak and war in Ukraine, we analyzed new HIV/AIDS diagnoses in three periods: pre-pandemic (March 2019 to February 2020), pandemic (March 2020 to May 2023), and war refugees from Ukraine migration period (February 2022 to September 2023). To analyze long-term trends, we used anonymous data from databases maintained by state entities available for public usage, i.e., the Epimeld database published by the National Institute of Public Health—National Research Institute (NIPH-NRI) [22]. These reports included suspected and confirmed HIV infection cases and HIV/AIDS diagnoses and were transferred to the District Sanitary and Epidemiological Station for verification and recording by the Polish administrative branch. Data from the Central Statistical Office on deaths related to HIV/AIDS were also used. The period for the analysis was chosen taking into account comparative data presented according to the same methodology. It started from 2009 and covered the subsequent calendar year until 2021, as the last year for which comparable infection and death data were available.

The possible influence of the COVID-19 pandemic to HIV/AIDS trends using monthly anonymous data from March 2020 to May 2023 was investigated and compared with pre-pandemic data from March 2019 to February 2020 (one calendar year before the announcement of the epidemic) [23]. The study also included new cases of HIV/AIDS diagnoses in the migration of the war refugees from Ukraine period (from February 2022 to September 2023) compared to the pre-pandemic period. Data by month on registered diagnosed cases of HIV/AIDS from March 2019 to September 2023 were received from epidemiological reports published by NIPH-NRI [23]. Information on the number of confirmed COVID-19 cases was obtained from the OurWorldData.org portal.

### 2.2. Study Variables

The analysis was conducted for newly diagnosed HIV/AIDS cases and COVID-19 recorded according to the International Statistical Classification of Diseases and Health Problems, Tenth Revision (ICD-10). The codes for HIV (Z21), HIV/AIDS (B20.0–B24), and COVID-19 (U07.1) cases were used in the study. The rate of new HIV diagnoses was calculated per 100,000 population for each calendar year from 2009 to 2021 (rates for total newly diagnosed cases of HIV per total population in Poland) with separate analysis for foreigners (rates for newly diagnosed cases of HIV among foreigners per total population in Poland). In addition, we calculated rates of new HIV diagnoses in 2011 and 2021 based on the national census (rates for total newly diagnosed cases of HIV per total population in Poland, rates for newly diagnosed cases of HIV among the Polish population per Polish population, and rates for newly diagnosed cases of HIV among foreigners per foreigners population in Poland). The rate of new HIV/AIDS diagnoses was calculated as the new cases of HIV/AIDS per 100,000 population in each calendar year over the 2009–2021 period, considering age groups (≤29, 30–39, 40–49, ≥50), gender, and transmission groups divided into men who have sex with men (MSM), persons who inject drugs (PWID), heterosexuals, mother-to-child transmission (MTCT), and unknown.

To estimate the extent of new HIV/AIDS cases during the COVID-19 pandemic, we examined the pre-pandemic period by considering the dominant viral variants associated with disease severity. In our study, the duration of the pandemic was divided into four periods: pre-Delta period (March 2020–June 2021), Delta SARS-CoV-2 dominance period (Delta period) (July 2021–December 2021), early Omicron SARS-CoV-2 variant dominance (with prevalence of BA.1 and BA.2 subvariants) (January 2022–June 2022), and late Omicron SARS-CoV-2 variant dominance (with BA.5, BQ.1, XBB, XBB.1.5, EG.5.1 prevalence) (July 2022–September 2023) [24,25]. In order to assess the impact of the migration of the refugees from the war in Ukraine on the epidemiology of HIV/AIDS in Poland, we investigated the rate trends of new HIV/AIDS cases by month over February 2022–September 2023. The rates of total new HIV/AIDS and COVID-19 diagnoses were calculated per 100,000 population per month.

We used age-standardized HIV/AIDS mortality rates (ASMRs) calculated using the direct standardization method and the European Standard Population in 2013 as the reference population. ASMR per 100,000 population was determined for each calendar year by age group (≤29, 30–39, 40–49, ≥50) and gender. Data on HIV/AIDS mortality were investigated as AIDS-associated deaths (B20–B22, B24) and non-AIDS associated (B23). In addition, mortalities due to AIDS-defining illness—tuberculosis (B20.0), pneumocystis carinii pneumonia (B20.6), non-Hodgkin lymphoma (B21.1–B21.2), dementia (B22.0), and interstitial pneumonitis (B22.1)—were analyzed.

### 2.3. Statistical Analysis

In order to investigate the long-term trends in new HIV/AIDS infections and mortality, and to analyze changes of trends from 2009 to 2021, joinpoint regression was applied [26,27]. Joinpoint regression identifies joinpoints that connect distinct line segments, enabling identification of years when a significant change in the linear slope of the trend (on a log scale) was obtained in data over time [26]. The study started with the minimum of joinpoints (e.g., zero joinpoints, which is a straight line) and used a maximum of two joinpoints (corresponding to three-line segments). Estimated linear segments were presented as an annual percentage change (APC) with a 95% confidence interval (CI). A summary of the 2009–2021 period revealed the annual average percentage change (AAPC) and its 95% CI, calculated as a weighted average of partial trend APCs [28]. The burden of new HIV/AIDS during the COVID-19 pandemic and in the period of migration of the war refugees from Ukraine was analyzed by the percentage change of the rates of new HIV/AIDS diagnoses.

Statistical data analysis was performed using IBM SPSS Software (version 24.0); data are statistically significant when *p* < 0.05.

## 3. Results

### 3.1. General Epidemiology of HIV/AIDS

From 2009 to 2021, a total of 15,410 new cases of HIV infection, 1798 new cases of HIV/AIDS, and 1460 deaths due to HIV/AIDS were diagnosed in Poland, with a higher prevalence among males (67.5% of HIV/AIDS diagnoses and 78.2% of deaths due to HIV/AIDS). From March 2020 until September 2023, 6203 new cases of HIV infection and 333 cases of new HIV/AIDS were detected. Most (69% of new HIV and 73% of new HIV/AIDS) were diagnosed after February 2022.

From 2009 to 2021, the median of new diagnoses for HIV and AIDS was 3.10 per 10^5^ and 0.35 per 10^5^, respectively, and the total ASMR of HIV/AIDS was 0.27 per 10^5^. An upward trend in new HIV diagnoses was seen when comparing 2021 vs. 2011 (by 6% in the total population and 3% among Poles). At the same time, a noticeable increase in new HIV diagnoses was observed for foreigners (by 73%) (Table 1).

### 3.2. Transmission Groups

**Figure 1:**
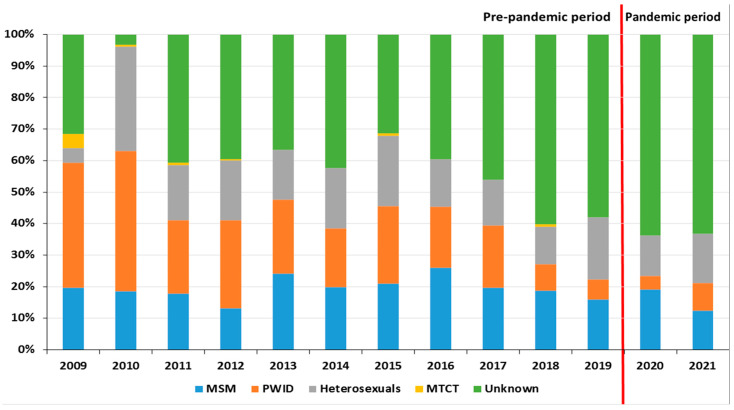
Distribution of HIV/AIDS transmission before and during the COVID-19 pandemic in Poland. Abbreviations: MSM—men who have sex with men, PWID—persons who inject drugs, MTCT—mother-to-child transmission.

The HIV transmission group in newly diagnosed HIV/AIDS cases was largely unknown, from 31 to 60% in 2011–2019 and increasing to 63–64% in the pandemic period. A large proportion of transmissions were associated with PWID in 2009–2012 (23–45%). In 2013–2017, the transmission percentage among PWID ranged from 19 to 24%, similar to among MSM (20 to 26%). For the remaining known transmission categories, the percentages in the pre-pandemic (2018–2019) and pandemic period (2020–2021) were for MSM—17% vs. 15%, heterosexuals—16% vs. 14%, and PWID—both 7%. A small proportion of transmission involved MTCT (1–4%), mainly in 2009–2012 (Figure 1).

### 3.3. Joinpoint Analysis of Long-Term Trends in New HIV/AIDS Diagnoses

**Figure 2:**
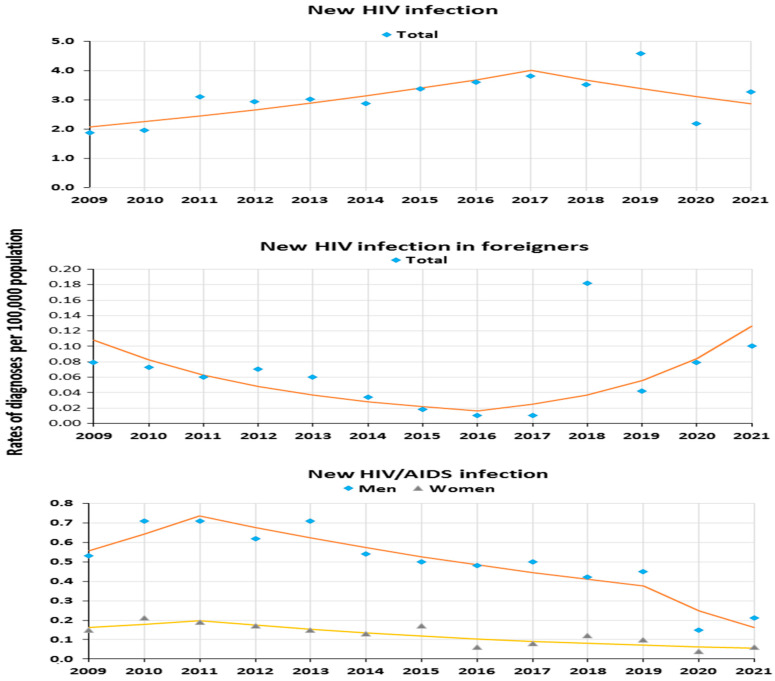
New HIV/AIDS infection in 2009–2021 in Poland as a trend modeled with joinpoint regression.

Trends of new HIV diagnoses showed rising changes in the total population (AAPC_2009–2021_ was +3.7%, *p* > 0.05) and decreasing changes among foreigners (AAPC_2009–2021_ was −0.9%, *p* > 0.05). However, the dynamic of partial trends in the total population differed from the trends among foreigners. For new HIV diagnoses, the first trend was increasing in the general population (APC_2009–2017_ +8.5%, *p* < 0.05), and in the second trend, there was a decrease (APC_2017–2021_ −7.9%, *p* > 0.05). Among foreigners, the first trend for new HIV diagnoses was negative (APC_2009–2016_ −23.6%, *p* > 0.05), and then the direction reversed to positive (APC_2016–2021_ +50.4%, *p* > 0.05) (Table 2 and Figure 2).

Changes in trends of new HIV/AIDS diagnosis in the total population were visible mainly at the two joinpoints connecting the three-line trend segment. For the total rate of new HIV/AIDS, the trend until 2011 was increasing (+22.5%, *p* > 0.05) to decrease later (APC_2011–2019_ −9.2%, *p* < 0.05). From 2019, a sharp decrease was observed (APC_2019–2021_ −30.7%, *p* < 0.05). The rate trends of the new HIV/AIDS diagnoses in men were similar to the changes in the total population, with a three-line pattern, contrary to those observed for women, who revealed a two-line trend (Table 2 and Figure 2).

Significant rate changes of new HIV/AIDS diagnoses were revealed for the age group 30–39 years, in which after 2017, the trend was significantly decreasing (APC_2017–2021_ was −27.1%). In other age groups (≤29, 40–49, ≥50), partial trends were generally decreasing and insignificant. Joinpoint analysis for transmission groups showed a significant decrease among heterosexuals (APC_2011–2019_ −12.9%, *p* < 0.05) and PWID (APC_2016–2021_ −36.1%, *p* < 0.05) (Table 2).

In the total analyzed period AAPC_2009–2021_, rates of new HIV/AIDS diagnoses assumed decreasing values, and these changes were visible in total and both genders (*p* < 0.05) except for the results in the oldest age group ≥ 50 (Table 2).

### 3.4. HIV/AIDS during the Periods of COVID-19 Pandemic and Refugees’ Migration from the War

**Figure 3:**
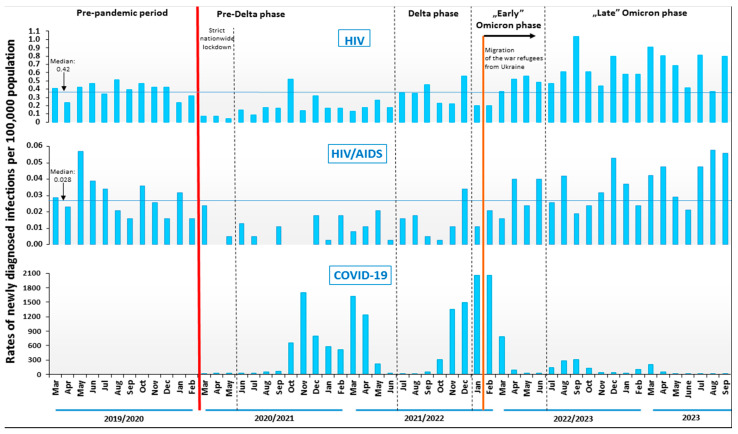
The burden of HIV/AIDS in Poland before and during the COVID-19 pandemic and during the migration of war refugees from Ukraine.

During the first 25 months of the COVID-19 pandemic (from March 2020 to March 2022), the rates of new HIV/AIDS diagnoses were lower than in the relative months of the pre-pandemic period. In the pre-Delta phase, the median rate of new HIV and HIV/AIDS diagnoses were 0.42 per 10^5^ and 0.28 per 10^5^, respectively (by −60% and 75% vs. the pre-pandemic period). Especially during the strict nationwide lockdown, there was a sharp decrease in rates of new HIV and HIV/AIDS diagnoses (median: 0.07 per 10^5^ and 0.005 per 10^5^, respectively). After this period, the median values of new HIV/AIDS rates increased in subsequent pandemic phases. In the Delta phase, the median for new HIV diagnoses was 0.36 per 10^5^ and 0.014 per 10^5^ for new HIV/AIDS, although their levels were lower than in the pre-pandemic period (by −14% and −50%, respectively). In the “early” Omicron phase, the median of new HIV diagnoses (0.43 per 10^5^) was similar to that in the pre-pandemic period, and the rate of new HIV/AIDS diagnoses was 0.023 per 10^5^ and was lower by 18%, respectively. During the “early” Omicron phase appeared the migration of war refugees from Ukraine, and this time, from April 2022, the rates of new HIV and new HIV/AIDS diagnoses increased in comparison to the pre-pandemic period. In the last phase of the pandemic, “late” Omicron rates of new HIV and new HIV/AIDS were 0.61 per 10^5^ and 0.037 per 10^5,^ and these values exceeded the level of rates achieved in the pre-pandemic period (by +45% and by +32%, respectively) (Figure 3).

### 3.5. Joinpoint Analysis of Long-Term Trends in Mortality Due to HIV/AIDS

**Figure 4:**
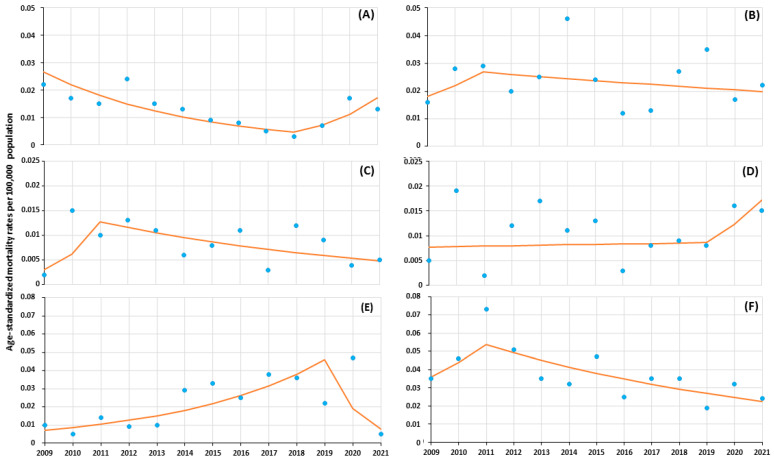
Mortality due to HIV/AIDS in 2009–2021 in Poland as a trend modeled with joinpoint regression, (**A**) tuberculosis, (**B**) pneumocystis carinii pneumonia, (**C**) non-Hodgkin lymphoma, (**D**) dementia, (**E**) interstitial pneumonitis, (**F**) non-AIDS-associated.

The analysis of mortality due to HIV/AIDS over 2009–2021 showed the changes with one joinpoint connecting two-line segments of the trend (Table 3 and Figure 4).

In the first trend over the period of 2009–2011, the value of ASMR increased among men, women, and in total, but changes for all were insignificant. In subsequent years until 2021, there was a significant decrease in ASMR among men (−2.8%/year), women (−6.5%/year), and in total (−3.6%/year). Similar marked changes in mortality during the second trend (2011–2021) were also visible in the age group 40–49 (−4.8%/year) and for non-AIDS associated mortality (−8.3%/year). Among the causes of mortality, two AIDS-defining illnesses showed a significant first trend with a change in direction during the second trend (for tuberculosis APC_2009–2018_ −17.5% and interstitial pneumonitis APC_2009–2019_ +20.3%), and in subsequent years in the case of both infectious diseases, there was a change in the direction of the trend, but it did not reach statistical significance.

## 4. Discussion

Our study was among the first to investigate the long-term trends in new HIV/AIDS infection and mortality in Poland using a joinpoint regression, considering various socio-demographic characteristics. It provides a comprehensive overview of the HIV/AIDS epidemiological situation in Poland over the last 14 years, exploring the influence of the COVID-19 pandemic outbreak and, more recently, a war in Ukraine and the subsequent migration of refugees. These observations have implications for the whole WHO European Region in achieving the global goal to end AIDS before 2030, but within which HIV transmission remains a significant concern [13,29].

The study demonstrates an apparent impact of the COVID-19 pandemic on new HIV/AIDS diagnoses, which declined sharply, especially during the national lockdown and pre-Delta period. Such an effect of public health strategies designed to limit the spread of SARS-CoV-2 on reduced access to HIV diagnostic services was already evidenced in other research, indicating that from one side, HIV transmission in the general population could be diminished due to decreased social mobility, but increased in a limited number of clusters of high-risk individuals [30,31,32]. In 2020 alone, an estimated million HIV diagnostic tests were not performed in Europe, and 25 thousand European inhabitants living with HIV were not diagnosed during the pandemic [33,34,35]. To understand how the situation evolved, we explored the subsequent pandemic phases, showing a rebound in novel HIV/AIDS cases, most likely due to the lifting of sanitary measures. The release of COVID-19 vaccines in 2021, appearance and transmission of the clinically less severe SARS-CoV-2 Omicron lineage in 2022, and declaring an end to COVID-19 as a Public Health Emergency Of International Concern [25] could result in an improved detection of HIV cases in various countries. In line with it, the highest increase in diagnostic rates was observed during the period dominated by “late” Omicron subvariants (BA.5, BQ1, and XBB sublineage), which were shown to be less severe than early ones (BA.1 and BA.2) [24]. Moreover, some studies found a significantly increased rate of HIV high-risk behavior in the post-lockdown era, especially among MSM [36,37]. Altogether, these turns may have significant consequences on HIV prevention in the considered region.

Moreover, the present study evidenced a concerning increase in new HIV cases among foreigners between 2011 and 2021, elevated further when a war started in Ukraine. Prior to the war, the HIV situation in Ukraine was already challenging, with only 69% of the estimated 260 thousand people aware of their infection status and 57% receiving ART [38]. In 2019–2021, HIV incidence was 40.6–42.5 per 100 thousand population, while the new cases constituted 12–15% of Europe’s total. Contrary to various other European populations in which infections are more common in men, Ukraine had a nearly equal distribution across both sexes [39]. In the first month of the war, 1.8 million people came to Poland from Ukraine. A year later, 1.4 million residents of Ukraine had a valid residence permit in Poland, of which the majority were women and children [40]. As previously shown, a war in Ukraine was associated with the cross-border inflow of HIV-infected women and introductions of the A6 HIV lineage [41,42]. Unfavorable HIV outcomes among foreigners living in Poland should drive health policies, including increased preventive activities encompassing education and diagnostics. Such measures should particularly target war refugees to put HIV transmission under better control.

Study limitations should be stressed. Firstly, HIV transmission among particular groups was not performed due to incomplete data, although HIV transmission in newly diagnosed HIV/AIDS cases was performed. Secondly, a relatively high proportion were categorized into unknown transmission routes, indicating that transmission among MSM may be particularly underestimated due to fears of stigma and homophobia, which remains a significant issue in Poland [43,44,45]. Moreover, it could not be established whether newly detected HIV infections in foreigners were previously detected in their native countries and whether these individuals received treatment (later abandoned due to war, migration, etc.). Even if this was the case, the detection of these infections in Poland is pivotal for the reintroduction of the appropriate treatment. It also indirectly indicates that numerous cases within groups of foreigners may remain undetected, stressing the need for broader monitoring. One should note that the Epimeld database applied in our research does not include the results obtained by the anonymous testing points, which operate in major Polish cities. Therefore, the observations of the present study, though important for understanding the HIV situation in Poland, may not fully reflect the scale of new HIV infections detected in the analyzed period. Of note, this calls for a discussion on the possible inclusion of anonymous testing points in the medical system. Last but not least, it is not known whether increased HIV/AIDS cases seen following an inflow of war refugees will impact the long-term epidemiological situation in the region, and no definitive conclusions should be drawn; these will be possible only if specific analysis of coming years will be conducted.

## 5. Conclusions

This study shows the increasing trend in HIV diagnoses in 2009–2017 in Poland, with a noticeable increase in the contribution of cases in foreigners. The diminished detection of new cases during earlier periods of the COVID-19 pandemic was followed by its rebound when restrictions were lifted, additionally fueled by the inflow of war refugees. Achieving the goal to eradicate AIDS as a public health hazard by 2030 requires Poland to engage more in active epidemiological surveillance, encompassing migrants and educational efforts.

## Figures and Tables

**Table 1:** Distribution of descriptive statistics for HIV/AIDS infection and mortality in Poland over the years 2009–2021.

Indicator	Median	Minimum	Maximum
	**Diagnosis rates per 100,000 population**		
New HIV diagnoses in			
Total (per total population in Poland)	3.10	1.88	4.59
Foreigners (per total population in Poland)	0.06	0.01	0.18
Total (per total population in Poland)	3.10 (2011 *) 3.28 (2021 *)	NA	NA
Polish population (per Polish population)	3.13 (2011 *) 3.22 (2021 *)	NA	NA
Foreigners (per foreigners population in Poland)	1.57 (2011 *) 2.71 (2021 *)	NA	NA
New HIV/AIDS diagnoses			
Men total	0.50	0.15	0.71
≤29	0.16	0.03	0.32
30–39	1.31	0.29	2.00
40–49	1.16	0.41	1.93
≥50	0.27	0.06	0.46
Women total	0.13	0.04	0.21
≤29	0.05	0.00	0.14
30–39	0.33	0.03	0.56
40–49	0.26	0.04	0.50
≥50	0.05	0.03	0.12
Total	0.35	0.12	0.63
≤29	0.10	0.02	0.23
30–39	0.72	0.17	1.28
40–49	0.71	0.24	1.10
≥50	0.15	0.06	0.25
Unknown	0.04	0.01	0.18
HIV transmission in newly diagnosed AIDS			
Men who have sex with men	0.07	0.02	0.12
Persons who inject drugs	0.07	0.01	0.18
Heterosexuals	0.06	0.02	0.14
Mother-to-child transmission	0.00	0.00	0.02
Unknown	0.14	0.01	0.25
	**Age-standardized mortality rates** **per 100,000 population**		
Mortality due to HIV/AIDS			
Men total	0.43	0.35	0.57
Women total	0.12	0.08	0.18
Total	0.27	0.23	0.35
≤29	0.02	0.03	0.03
30–39	0.08	0.05	0.12
40–49	0.11	0.08	0.15
≥50	0.06	0.04	0.09
AIDS-associated mortality	0.23	0.19	0.31
Non-AIDS associated mortality	0.04	0.02	0.07
Mortality due to AIDS-defining illness			
Tuberculosis	0.01	0.003	0.02
Pneumocystis carinii pneumonia	0.02	0.01	0.05
Non-Hodgkin lymphoma	0.01	0.002	0.02
Dementia	0.01	0.002	0.02
Interstitial pneumonitis	0.02	0.01	0.05

Abbreviations: AIDS—acquired immune deficiency syndrome, HIV—human immunodeficiency virus, NA—not applicable, * rates based on the national census in 2021 and 2021, respectively.

**Table 2:** Trends of new HIV/AIDS infection by gender, age, and transmission groups in 2009–2021 in Poland.

Indicators HIV/AIDS	Joinpoint Analysis to Identify Changes in Trends over 2009–2021						AAPC
	The Trend for Period 1	APC	The Trend for Period 2	APC	The Trend for Period 3	APC	
**New HIV diagnoses**							
Total	2009–2017	8.5 * **(0.8, 16.8)**	2017–2021	−7.9 (−25.6, 13.9)			3.7 (−0.1, 7.7)
Foreigners	2009–2016	−23.6 (−43.7, 3.7)	2016–2021	50.4 (−9.7, 150.6)			−0.9 (−14.6, 15.1)
**New HIV/AIDS diagnoses**							
MEN							
Total	2009–2011	14.9 (−17.0, 59.1)	2011–2019	−8.0 * **(−11.9, −3.9)**	2019–2021	−34.2 * **(−52.5, −8.9)**	−9.0 * **(−13.3, −4.4)**
≤29	2009–2019	−7.9 * **(−15.1, −0.1)**	2019–2021	−57.3 (−84.9, 21.2)			−14.3 * **(−21.1, −6.9)**
30–39	2009–2016	−3.2 (−14.8, 10.0)	2016–2021	−25.6 * **(−40.0, −7.9)**			−12.5 * **(−17.6, −7.2)**
40–49	2009–2018	−4.3 (−11.0, 3.0)	2018–2021	−21.5 (−47.3, 17.0)			−7.5 * **(−11.4, −3.5)**
≥50	2009–2011	42.7 (−77.6, 807.4)	2011–2018	−3.4 (−29.4, 32.0)	2018–2021	−31.7 (−72.9, 72.2)	−5.8 (−13.9, 3.1)
WOMEN							
Total	2009–2011	10.4 (−63.8, 237.1)	2011–2021	−12.0 * **(−19.3, −4.0)**			−10.1 * **(−14.7, −5.3)**
≤29	2009–2019	−16.6 * **(−24.6, −7.7)**	NA				NA
30–39	2009–2016	−19.0 (−36.8, 3.7)	2016–2021	−8.8 (−39.7, 38.0)			−15.2 * **(−25.5, −3.6)**
40–49	2009–2017	−11.5 (−22.9, 1.6)	2017–2021	−6.5 (−37.3, 39.4)			−10.1 * **(−16.8, −3.0)**
≥50	2009–2012	13.7 (−45.1, 135.5)	2012–2021	−8.1 (−19.5, 5.0)			−4.6 (−10.8, 2.1)
TOTAL	2009–2011	22.5 (−10.0, 66.7)	2011–2019	−9.2 * **(−12.9, −5.4)**	2019–2021	−30.7 * **(−49.1, −5.7)**	−9.0 * **(−13.1, −4.6)**
≤29	2009–2014	−5.3 (−26.2, 21.5)	2014–2019	−13.7 (−39.3, 22.8)	2019–2021	−52.3 (−84.4, 45.4)	−15.4 * **(−21.4, −8.8)**
30–39	2009–2017	−6.4 (−15.2, 3.4)	2017–2021	−27.1 * **(−45.4, −2.7)**			−12.6 * **(−17.1, −7.8)**
40–49	2009–2012	5.0 (−17.9, 34.3)	2012–2019	−7.7 (−15.1, 0.3)	2019–2021	−25.0 (−54.2, 22.7)	−7.4 * **(−11.4, −3.2)**
≥50	2009–2011	34.8 (−51.9, 277.8)	2011–2019	−4.8 (−17.0, 9.3)	2019–2021	−37.1 (−77.6, 76.1)	−5.4 (−11.8, 1.5)
Transmission groups							
MSM	2009–2013	8.6 (−14.4, 37.7)	2013–2019	−10.9 (−24.7, 5.4)	2019–2021	−42.1 (−72.7, 22.6)	−9.6 * **(−15.0, −3.9)**
PWID	2009–2016	−12.6 (−26.8, 4.3)	2016–2021	−36.1 * **(−52.4, −14.2)**			−22.5 * **(−28.2, −16.4)**
Heterosexuals	2009–2011	24.6 (−22.2, 99.6)	2011–2019	−12.9 * **(−18.3, −7.3)**	2019–2021	−29.4 (−55.9, 13.1)	−11.7 * **(−17.0, −6.2)**

Abbreviations: APC—annual percent change, AAPC—average annual percent change for total study period 2009–2021, AIDS—acquired immune deficiency syndrome, HIV—human immunodeficiency virus, NA—not applicable due to lack of AIDS cases in some years between 2013 and 2021, MSM—men who have sex with men, PWID—persons who inject drugs. *—Statistically significant trend at *p* < 0.05 (in bold).

**Table 3:** Trends of age-standardized mortality rates due to HIV/AIDS in Poland over the years 2009–2021.

Mortality Due to HIV/AIDS	Joinpoint Analysis to Identify Changes in Trends over 2009–2021				AAPC
	Trend for Period 1	APC	Trend for Period 2	APC	
Men	2009–2011	12.3 (−18.2, 54.2)	2011–2021	−2.8 * **(−5.2, −0.4)**	–1.5 (–3.6, 0.7)
Women	2009–2011	21.5 (–45.7, 172.2)	2011–2021	−6.5 * **(−12.2, −0.4)**	−4.1 * **(−8.0, −0.1)**
Total	2009–2011	14.2 (–22.1, 67.4)	2011–2021	−3.6 * **(−6.4, −0.7)**	–2.0 (–4.3, 0.2)
≤29	2009–2019	–2.7 (–8.3, 3.4)	2019–2021	3.0 (–52.5, 123.0)	–2.1 (–6.1, 2.0)
30–39	2009–2018	−6.4 * **(−12.4, −0.1)**	2018–2021	5.4 (–26.8, 51.6)	−4.5 * **(−7.8, −1.0)**
40–49	2009–2011	19.5 (–31.7, 108.9)	2011–2021	−4.8 * **(−8.9, −0.6)**	–2.8 (–6.1, 0.7)
≥50	2009–2014	13.6 * **(1.2, 27.5)**	2014–2021	–3.4 (–9.9, 3.5)	2.8 (–0.7, 6.4)
AIDS-associated mortality	2009–2011	12.0 (–26.6, 71.0)	2011–2021	–2.8 (–5.9, 0.5)	–1.5 (–3.8, 0.9)
Non-AIDS-associated mortality	2009–2011	22.2 (–44.4, 168.4)	2011–2021	−8.3 * **(−13.7, −2.5)**	−5.8 * **(−9.9, −1.4)**
Mortality due to AIDS-defining illness					
Tuberculosis	2009–2018	−17.5 * **(−24.2, −10.1)**	2018–2021	53.3 (–4.1, 145.0)	–8.0 (–15.6, 0.4)
Pneumocystis carinii pneumonia	2009–2011	21.9 (–70.6, 405.2)	2011–2021	–3.0 (–13.1, 8.4)	–0.9 (–7.1, 5.8)
Non-Hodgkin lymphoma	2009–2011	103.3 (–55.4, 826.2)	2011–2021	–9.1 (–19.3, 2.2)	–2.0 (–11.8, 8.7)
Dementia	2009–2019	1.1 (–17.2, 23.4)	2019–2021	41.2 (–89.1, 172.6)	4.3 (–6.9, 16.8)
Interstitial pneumonitis	2009–2019	20.3 * **(9.2, 32.6)**	2019–2021	–58.6 (–88.1, 44.1)	8.9 (–3.3, 22.7)

Abbreviations: APC—annual percent change, AAPC—average annual percent change for total study period 2009–2021, AIDS—acquired immune deficiency syndrome, HIV—human immunodeficiency virus, *—statistically significant trend at *p* < 0.05 (in bold).

## Data Availability

Data were collected from publicly archived Polish administrative registers (Epimeld and Central Statistical Office), and the OurWorldData.org portal.
